# 3-Meth­oxy-3-oxopropanaminium chloride

**DOI:** 10.1107/S1600536812003297

**Published:** 2012-02-04

**Authors:** Tobias Gruber, Christopher J. Schofield, Amber L. Thompson

**Affiliations:** a Chemistry Research Laboratory, Department of Chemistry, University of Oxford, Mansfield Road, Oxford OX1 3TA, England; b Chemical Crystallography, Chemistry Research Laboratory, Department of Chemistry, University of Oxford, Mansfield Road, Oxford OX1 3TA, England

## Abstract

In the title compound, C_4_H_10_NO_2_^+^·Cl^−^, the central ethyl­ene bond of the cation adopts a *gauche* conformation. The three H atoms of the –NH_3_^+^ group are engaged in strong and highly directional inter­molecular N—H⋯Cl hydrogen bonds, which result in a tape-like arrangement along [010] of the respective ion pairs. In addition, weak inter­molecular C—H⋯Cl and C—H⋯O inter­actions are present.

## Related literature

For the synthesis of the title compound, see: Hansen (1963 ▶). For related structures, see: Akkerman *et al.* (2003 ▶); Robinson *et al.* (2004 ▶); Vilela *et al.* (2009 ▶); Tarafdar & Swamy (2010 ▶); Gossage *et al.* (2010 ▶); He *et al.* (2010 ▶). For information on the *gauche* effect, see: Amos *et al.* (1992 ▶). For details of the H-atom treatment, see: Cooper *et al.* (2010 ▶). For the weighting scheme used in the refinement, see: Watkin (1994 ▶); Prince (1982 ▶).
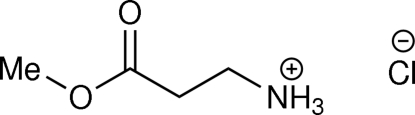


## Experimental

### 

#### Crystal data

C_4_H_10_NO_2_^+^·Cl^−^
*M* *_r_* = 139.58
Monoclinic, 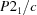

*a* = 9.8469 (2) Å
*b* = 5.3263 (1) Å
*c* = 13.2804 (2) Å
β = 99.4638 (10)°
*V* = 687.04 (2) Å^3^
*Z* = 4
Mo *K*α radiation
μ = 0.47 mm^−1^
*T* = 150 K
0.28 × 0.13 × 0.08 mm

#### Data collection

Nonius KappaCCD diffractometer
Absorption correction: multi-scan *DENZO*/*SCALEPACK* (Otwinowski & Minor, 1997 ▶) *T*_min_ = 0.94, *T*_max_ = 0.96
14336 measured reflections
1563 independent reflections
1413 reflections with *I* > 2σ(*I*)
*R*_int_ = 0.014

#### Refinement

*R*[*F* ^2^ > 2σ(*F* ^2^)] = 0.032
*wR*(*F* ^2^) = 0.080
*S* = 0.93
1563 reflections
73 parameters
H-atom parameters constrained
Δρ_max_ = 0.25 e Å^−3^
Δρ_min_ = −0.28 e Å^−3^

### 

Data collection: *COLLECT* (Nonius, 2001 ▶).; cell refinement: *DENZO* and *SCALEPACK* (Otwinowski & Minor, 1997 ▶); data reduction: *DENZO* and *SCALEPACK*; program(s) used to solve structure: *SIR92* (Altomare *et al.*, 1994 ▶); program(s) used to refine structure: *CRYSTALS* (Betteridge *et al.*, 2003 ▶); molecular graphics: *CAMERON* (Watkin *et al.*, 1996 ▶); software used to prepare material for publication: *CRYSTALS* and *PLATON* (Spek, 2009 ▶).

## Supplementary Material

Crystal structure: contains datablock(s) I, global. DOI: 10.1107/S1600536812003297/lh5384sup1.cif

Structure factors: contains datablock(s) I. DOI: 10.1107/S1600536812003297/lh5384Isup2.hkl

Supplementary material file. DOI: 10.1107/S1600536812003297/lh5384Isup3.cdx

Supplementary material file. DOI: 10.1107/S1600536812003297/lh5384Isup4.cml

Additional supplementary materials:  crystallographic information; 3D view; checkCIF report

## Figures and Tables

**Table 1:** Hydrogen-bond geometry (Å, °)

*D*—H⋯*A*	*D*—H	H⋯*A*	*D*⋯*A*	*D*—H⋯*A*
N8—H81⋯Cl1^i^	0.90	2.26	3.1456 (12)	171 (1)
N8—H82⋯Cl1	0.92	2.29	3.1910 (12)	171 (1)
N8—H83⋯Cl1^ii^	0.90	2.35	3.1923 (12)	157 (1)
C5—H53⋯O4^iii^	0.96	2.67	3.5965 (18)	163 (1)
C7—H72⋯Cl1^iv^	0.96	2.84	3.4708 (14)	124 (1)

Symmetry codes: (i) 
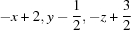
; (ii) 
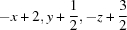
; (iii) 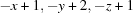; (iv) 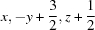.

**Fig. 1.**
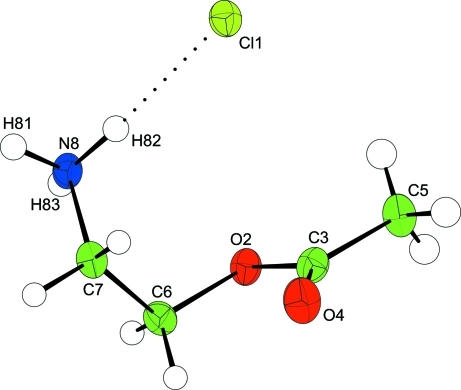
Molecular structure of (I) with displacement ellipsoids drawn at 50% probability. The dotted line indicates a hydrogen bond.

**Fig. 2.**
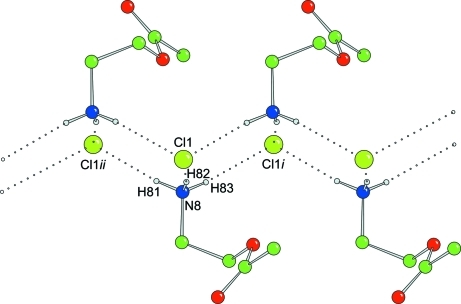
The corrugated two dimensional supramolecular network defined by the N—H···Cl interactions forming tapes [i: 2 - x,1/2 + y,3/2 - z; ii: 2 - x,-1/2 + y,3/2 - z].

## supplementary crystallographic information

### Comment

The asymmetic unit of the title compound, (I), consists of a 2-acetoxy-ethyl-ammonium cation and a chloride ion as shown in Figure 1. The ester motif [atoms C6/O2/C3/O4/C5] is approximately planar with the largest deviation from the mean plane for O2 (*d* = 0.029 Å). The central —CH_2_—CH_2_— unit is not in the often favoured antiperiplanar conformation, instead adopting a *gauche* conformation with a torsion angle of 57.42 (14)° for atoms O2—C6—C7—N8. This may be attributed to the stereoselective *gauche* effect (Amos *et al.*, 1992), though an influence of the crystal packing on the molecular conformation of (I) cannot be ruled out. For comparison, the observed torsion angle is 67.6° in 1,2-difluoroethane (Akkerman *et al.*, 2003), 73.7° for *O*-stearoylethanolamine hydrochloride (Tarafdar & Swamy, 2010) and 71.7° in 2-(benzoyloxy)ethanaminium nitrate (Gossage *et al.*, 2010).

The three N—H units of (I) are engaged in apparently strong and highly directional N^+^—H···Cl^-^ hydrogen bonds with three symmetry-related Cl^-^ ions (Table 1). These interactions result in a tape-like arrangement of the respective ion pairs parallel to the crystallographic *b* axis (Figure 2). In the packing, the corrugated two dimensional supramolecular network defined by the N—H···Cl interactions is connected with neighbouring strands *via* weak C—H···Cl and C—H···O contacts (Table 1) in the direction of the crystallographic *c* and *a* axes, respectively. Interestingly, the observed packing behaviour is very similar to the structure of glycine ethyl ester hydrochloride (He *et al.*, 2010), an isomer of (I), and the analogous glycine methyl ester (Vilela *et al.*, 2009).

### Experimental

The title compound was prepared from 2-aminoethanol and acetyl chloride according to the literature (Hansen, 1963). Crystals suitable for X-ray diffraction were obtained by slow evaporation of a solution of (I) in chloroform.

### Refinement

The structure was refined freely, except for the hydrogen atoms which were refined prior to the generation of the riding model (Cooper *et al.*, 2010). Weights were applied using a five parameter Chebychev polynomial (Watkin, 1994, Prince, 1982).

Dihedral angles calculated with *PLATON* (Spek, 2009); all other standard uncertainties calculated from the full variance co-variance matrix within *CRYSTALS* (Betteridge *et al.*, 2003).

### Crystal data

**Table:**

C_4_H_10_NO_2_^+^·Cl^−^	*F*(000) = 296
*M**_r_* = 139.58	*D*_x_ = 1.349 Mg m^−^^3^
Monoclinic, *P*2_1_/*c*	Mo *K*α radiation, λ = 0.71073 Å
Hall symbol: -P 2ybc	Cell parameters from 1729 reflections
*a* = 9.8469 (2) Å	θ = 5–27°
*b* = 5.3263 (1) Å	µ = 0.47 mm^−^^1^
*c* = 13.2804 (2) Å	*T* = 150 K
β = 99.4638 (10)°	Block, clear_pale_colourless
*V* = 687.04 (2) Å^3^	0.28 × 0.13 × 0.08 mm
*Z* = 4	

### Data collection

**Table:**

Nonius KappaCCD diffractometer	1413 reflections with *I* > 2σ(*I*)
Graphite monochromator	*R*_int_ = 0.014
ω scans	θ_max_ = 27.5°, θ_min_ = 5.2°
Absorption correction: multi-scan *DENZO*/*SCALEPACK* (Otwinowski & Minor, 1997)	*h* = −12→12
*T*_min_ = 0.94, *T*_max_ = 0.96	*k* = −6→6
14336 measured reflections	*l* = −17→17
1563 independent reflections	

### Refinement

**Table:**

Refinement on *F*^2^	Primary atom site location: structure-invariant direct methods
Least-squares matrix: full	Hydrogen site location: difference Fourier map
*R*[*F*^2^ > 2σ(*F*^2^)] = 0.032	H-atom parameters constrained
*wR*(*F*^2^) = 0.080	Method, part 1, Chebychev polynomial, (Watkin, 1994; *P*rince, 1982) [*w*eight] = 1.0/[A_0_*T_0_(x) + A_1_*T_1_(x) ··· + A_n-1_]*T_n-1_(x)] where A_i_ are the Chebychev coefficients listed belo*w* and x = *F* /*F*max Method = Robust Weighting (*P*rince, 1982) W = [*w*eight] * [1-(delta*F*/6*sigma*F*)^2^]^2^ A_i_ are: 37.6 62.5 38.0 16.9 4.31
*S* = 0.93	(Δ/σ)_max_ = 0.001
1563 reflections	Δρ_max_ = 0.25 e Å^−^^3^
73 parameters	Δρ_min_ = −0.28 e Å^−^^3^
0 restraints	

### Fractional atomic coordinates and isotropic or equivalent isotropic displacement parameters (Å^2^)

**Table:**

	*x*	*y*	*z*	*U*_iso_*/*U*_eq_	
Cl1	0.92411 (3)	0.72604 (6)	0.59816 (2)	0.0244	
O2	0.72167 (10)	1.11170 (19)	0.75243 (7)	0.0236	
C3	0.62577 (14)	1.0662 (3)	0.66935 (11)	0.0241	
O4	0.53381 (11)	0.9165 (2)	0.66871 (8)	0.0335	
C5	0.64993 (16)	1.2257 (3)	0.58178 (12)	0.0307	
C6	0.70489 (15)	0.9789 (3)	0.84455 (10)	0.0257	
C7	0.76188 (14)	0.7166 (3)	0.84666 (10)	0.0240	
N8	0.91018 (12)	0.7208 (2)	0.83652 (9)	0.0235	
H51	0.7403	1.1944	0.5697	0.0451*	
H52	0.6422	1.3972	0.6003	0.0448*	
H53	0.5842	1.1883	0.5224	0.0448*	
H61	0.7569	1.0756	0.8995	0.0291*	
H62	0.6067	0.9749	0.8523	0.0288*	
H71	0.7136	0.6156	0.7909	0.0287*	
H72	0.7551	0.6399	0.9112	0.0286*	
H81	0.9506	0.5763	0.8594	0.0342*	
H82	0.9161	0.7420	0.7690	0.0341*	
H83	0.9517	0.8494	0.8726	0.0346*	

### Atomic displacement parameters (Å^2^)

**Table:**

	*U*^11^	*U*^22^	*U*^33^	*U*^12^	*U*^13^	*U*^23^
Cl1	0.02820 (19)	0.02258 (18)	0.02136 (18)	−0.00083 (12)	0.00108 (12)	−0.00105 (12)
O2	0.0248 (5)	0.0223 (5)	0.0222 (5)	−0.0002 (4)	−0.0009 (4)	0.0002 (4)
C3	0.0238 (6)	0.0228 (6)	0.0243 (6)	0.0016 (5)	−0.0005 (5)	−0.0012 (5)
O4	0.0303 (5)	0.0348 (6)	0.0329 (6)	−0.0080 (5)	−0.0025 (4)	0.0039 (5)
C5	0.0332 (8)	0.0305 (8)	0.0268 (7)	−0.0027 (6)	0.0000 (6)	0.0047 (6)
C6	0.0292 (7)	0.0278 (7)	0.0200 (6)	0.0021 (6)	0.0041 (5)	−0.0010 (5)
C7	0.0268 (7)	0.0229 (6)	0.0215 (6)	−0.0016 (5)	0.0017 (5)	0.0021 (5)
N8	0.0287 (6)	0.0202 (5)	0.0208 (5)	0.0032 (4)	0.0015 (4)	0.0012 (4)

### Geometric parameters (Å, º)

**Table:**

O2—C3	1.3509 (16)	C6—H61	0.968
O2—C6	1.4459 (17)	C6—H62	0.989
C3—O4	1.2057 (18)	C7—N8	1.4886 (18)
C3—C5	1.490 (2)	C7—H71	0.973
C5—H51	0.945	C7—H72	0.961
C5—H52	0.952	N8—H81	0.896
C5—H53	0.955	N8—H82	0.915
C6—C7	1.504 (2)	N8—H83	0.895
			
C3—O2—C6	116.20 (11)	C7—C6—H62	110.2
O2—C3—O4	123.21 (13)	H61—C6—H62	109.8
O2—C3—C5	110.81 (12)	C6—C7—N8	110.65 (11)
O4—C3—C5	125.98 (13)	C6—C7—H71	111.4
C3—C5—H51	107.9	N8—C7—H71	107.7
C3—C5—H52	108.4	C6—C7—H72	109.3
H51—C5—H52	109.3	N8—C7—H72	107.5
C3—C5—H53	110.6	H71—C7—H72	110.2
H51—C5—H53	110.7	C7—N8—H81	110.1
H52—C5—H53	109.9	C7—N8—H82	108.2
O2—C6—C7	112.08 (11)	H81—N8—H82	110.0
O2—C6—H61	104.9	C7—N8—H83	109.3
C7—C6—H61	109.3	H81—N8—H83	109.7
O2—C6—H62	110.3	H82—N8—H83	109.5

### Hydrogen-bond geometry (Å, º)

**Table:**

*D*—H···*A*	*D*—H	H···*A*	*D*···*A*	*D*—H···*A*
N8—H81···Cl1^i^	0.90	2.26	3.1456 (12)	171 (1)
N8—H82···Cl1	0.92	2.29	3.1910 (12)	171 (1)
N8—H83···Cl1^ii^	0.90	2.35	3.1923 (12)	157 (1)
C5—H53···O4^iii^	0.96	2.67	3.5965 (18)	163 (1)
C7—H72···Cl1^iv^	0.96	2.84	3.4708 (14)	124 (1)

Symmetry codes: (i) −*x*+2, *y*−1/2, −*z*+3/2; (ii) −*x*+2, *y*+1/2, −*z*+3/2; (iii) −*x*+1, −*y*+2, −*z*+1; (iv) *x*, −*y*+3/2, *z*+1/2.
